# A Hybrid Approach for IoT Security: Combining Ensemble Learning with Fuzzy Logic

**DOI:** 10.3390/s25185668

**Published:** 2025-09-11

**Authors:** Aykut Karakaya

**Affiliations:** Department of Computer Engineering, Zonguldak Bulent Ecevit University, 67100 Zonguldak, Türkiye; aykut.karakaya@beun.edu.tr

**Keywords:** IoT security, ensemble learning, fuzzy logic, malware detection

## Abstract

The rapid expansion of Internet of Things (IoT) devices has led to substantial progress in various fields. The diverse and resource-limited characteristics of IoT devices make them susceptible to numerous cyber threats, especially malware. Traditional security approaches fall short of effectively addressing these challenges. In this paper, a novel hybrid approach based on the integration of ensemble learning and fuzzy logic is proposed to enhance IoT security. While the ensemble learning model combines multiple classifiers to improve detection accuracy, fuzzy logic enables a more flexible and interpretable assessment of the security status of IoT systems. Experimental results reveal that the proposed framework provides high-accuracy malware detection and, through the fuzzy system built upon the rule base derived from the ensemble model, offers a more flexible and human intuition-oriented evaluation capability. This study offers an effective solution for ensuring IoT system security, providing an applicable approach across diverse IoT ecosystems.

## 1. Introduction

The rapid proliferation of IoT devices has led to significant transformations across various sectors, from smart homes to healthcare services and industrial automation. However, this also renders IoT devices vulnerable to various cyberattacks, due to their frequent lack of robust security measures. Considering the heterogeneity and scalability of IoT environments, traditional security approaches fall short in addressing the security needs of IoT systems [1]. This situation necessitates the development of innovative solutions capable of adapting to evolving threats. Recent studies have revealed that leveraging methods such as machine learning and ensemble learning to gain insights from large datasets is highly effective in detecting and mitigating IoT malware.

This paper proposes a hybrid framework that combines ensemble learning and fuzzy logic to assess IoT security. Leveraging an ensemble learning model trained on an IoT malware dataset, rules are automatically generated and then utilized within a fuzzy logic system to evaluate the security status of IoT networks. Unlike the strict outputs of traditional classification models, fuzzy logic provides a more detailed assessment by categorizing security status into three levels: secure, partially_insecure, and insecure. This approach enhances the accuracy of threat detection and delivers more comprehensive feedback. Furthermore, the integration of ensemble learning with fuzzy logic emerges as a promising direction in cybersecurity research. In fuzzy logic systems, the challenging process of defining the rule base is automated through ensemble learning. By combining ensemble learning models with fuzzy logic, decisions regarding the security status of newly acquired IoT system data can be made, thereby improving the overall resilience of IoT systems. In this context, the proposed framework addresses a important issue in IoT security by uniting the strengths of ensemble learning and fuzzy logic. By automating the rule-generation process and employing a flexible evaluation mechanism, this approach offers an effective solution adaptable to various IoT ecosystems.

### 1.1. Related Works

In recent years, IoT technology has become an indispensable part of modern life, driven by its rapidly expanding range of applications. This growth has also introduced various challenges for the efficient and secure operation of IoT systems. Critical issues such as security threats and routing strategies have gained greater importance with the widespread adoption of IoT. To detect and prevent attacks within a system, continuous monitoring is required, and research efforts in this area have increased in recent years [2]. A common theme in these studies is the demonstration of how advanced technologies such as fuzzy logic and machine learning play a critical role in enhancing the security, monitoring, and efficiency of IoT systems. In this context, the approach proposed in [3] to improve security in Cognitive IoT systems increased web spam detection accuracy to 97.3% by employing fuzzy rule-based classifiers and machine learning classifiers. Similarly, the secure and intelligent fuzzy blockchain framework developed in [4] for intrusion detection in IoT networks demonstrated superior performance in terms of accuracy, precision, recall, and F1-score. These studies highlight the effectiveness of applying fuzzy logic and machine learning techniques to address security issues in IoT systems. In IoT systems where these two concepts are used together, energy efficiency and routing emerge as critical research areas. In [5], a method combining fuzzy logic and reinforcement learning was proposed to provide reliable routing in IoT networks, improving network lifetime and energy efficiency compared to traditional protocols. Similarly, ref. [6] developed a fuzzy logic and trust-based routing protocol to enhance network performance and intrusion detection in mobile environments and large-scale networks.

Fuzzy logic also plays a significant role in cloud and fog-based IoT applications. In [7], interval-valued intuitionistic fuzzy analytical hierarchy process (IVIFS-AHP) was employed to evaluate security attributes, enabling the prioritization of security factors. In [8], a secure architecture for IoT security was proposed using fuzzy logic and fog computing, providing real-time detection of DDoS and collaborative attacks. These studies demonstrate the impact of fuzzy logic in enhancing security and data integrity in cloud- and fog-based IoT applications.

Detecting malicious behavior in IoT systems is another important issue. In [9], attack detection accuracy in IoT networks was improved by using software-defined networking and a fuzzy neural network. In [10], a general and lightweight security mechanism was developed to detect malicious attacks such as black hole and DDoS in uncertain IoT environments. This mechanism was reported to provide high accuracy, scalability, and low resource overhead. In [11], a fuzzy logic-based risk assessment model for industrial IoT security was developed, offering a security risk assessment and enabling effective solutions in decision-making processes.

In recent years, fuzzy logic-based approaches have attracted attention in studies aimed at detecting security vulnerabilities in IoT and WSN networks. Integrated with various machine learning techniques, these systems not only improve detection accuracy but also enhance network performance. In this context, proposed hybrid systems operate in conjunction with decision trees, clustering algorithms, and fuzzy inference mechanisms, achieving high success rates in intrusion detection and classification processes [12]. In addition, some studies have developed algorithms that aim to isolate malicious nodes and reduce retransmissions while maintaining energy efficiency [13]. Supported by deep learning and entropy-based methods, these approaches deliver effective results, particularly in real-time threat detection and secure data transmission [14].

Overall, these studies in the IoT domain demonstrate that fuzzy logic and related technologies play a significant role in addressing fundamental challenges such as security and routing. In addition, they reveal how advanced techniques such as fuzzy logic, machine learning, and ensemble learning can be effectively utilized to enhance the security, efficiency, and sustainability of IoT systems. Thus, these works contribute to the development of more secure, efficient, and sustainable systems in the future evolution of IoT technologies. The details of the reviewed articles are presented in Table 1.

In this paper, a hybrid framework that combines ensemble learning and fuzzy logic is proposed to analysis IoT security. An ensemble learning model trained on an IoT malware dataset automatically generates rules, which are then utilized in a fuzzy logic system for security evaluation. While traditional classification models produce definitive results, fuzzy logic provides more realistic and flexible outcomes for assessing security status. This approach enhances the accuracy of threat detection, and through the integration of ensemble learning and fuzzy logic, makes IoT security evaluation more effective. Furthermore, it offers a flexible and efficient solution adaptable to various IoT ecosystems.

### 1.2. Motivation and Contributions

The heterogeneous nature and resource constraints of IoT devices make them vulnerable to complex and constantly evolving cyber threats. Traditional security solutions fall short in detecting these threats both accurately and flexibly. In particular, binary classification-based approaches fail to capture intermediate states such as when a system is partially insecure, leading to incomplete evaluations in real-world scenarios. The primary motivation of this study is to develop a flexible, interpretable, and adaptable framework for assessing IoT security by combining machine learning-based models, which provide high accuracy, with fuzzy logic systems, which are better at handling uncertainties.

This paper offers the following key contributions:

- A majority voting-based ensemble architecture integrating multiple machine learning algorithms presents an innovative hybrid framework for malware detection and assessment in IoT environments.
- The automatically generated rule base derived from the high-accuracy labeling outputs of the ensemble model eliminates the need for manual rule definition in the fuzzy inference system, thereby enhancing the scalability and consistency of the process.
- A three-level security status modeling that goes beyond binary classification approaches is expressed through membership degrees, providing a more accurate representation of risks in gray areas. Unlike traditional machine learning or fixed threshold-based methods, this approach offers decision-makers a more flexible and human intuition-aligned assessment.
- The comprehensive dataset, encompassing realistic IoT network behaviors, various device types, and different attack scenarios, supports the applicability of the method under real-world conditions.

### 1.3. Organization

The rest of the paper is structured as follows: Section 2 presents the background information on the key methods and techniques used in the development of the proposed model. Section 3 details the proposed method, starting with the data-preprocessing steps and continuing with ensemble learning-based rule extraction and fuzzy inference system for determining the security status. Section 4 covers the experimental setup, performance evaluation metrics, results obtained, and discussions. Finally, Section 5 presents the overall conclusion of the paper.

## 2. Preliminaries

This section offers an overview of the methods used in developing the proposed model, serving as a preliminary finding.

### 2.1. SelectKBest Feature Selection

SelectKBest is a method used in feature selection that evaluates the relationship between features and the target variable through univariate statistical tests. This technique aims to reduce the dataset’s dimensionality by selecting a specified number of the best features (K-best). The scores calculated for each feature in this process reflect its relationship with the target variable. Features with the highest scores are considered to have the most influence on the target variable and are therefore selected [15]. In this paper, the feature-selection process was carried out using the SelectKBest method. The Analysis of Variance (ANOVA) F-value, often preferred when working with continuous data, was applied to determine the K-best features. The ANOVA F-test calculates the F-score for each feature by comparing the variance between independent variables and the dependent variable. This score is obtained by dividing the between-group variance by the within-group variance. A high F-score indicates that the associated feature has a strong effect on the target variable and is therefore selected. The ANOVA F-test is particularly effective in classification tasks, as it allows for the elimination of irrelevant or low-impact features in the dataset, thus improving the overall performance of the model. The formula for the ANOVA F-score is provided in Equation (1)

(1) $$F=\frac{{\mathrm{MS}}_{\mathrm{between}\;}}{{\mathrm{MS}}_{\mathrm{within}\;}}$$

Here, ${\mathrm{MS}}_{\mathrm{between}\;}$ represents the variance between groups (Mean Square Between), ${\mathrm{MS}}_{\mathrm{within}\;}$ represents the variance within groups (Mean Square Within).

The F-value is obtained by dividing the variance between groups by the variance within groups. A high F-score indicates that the group has a strong effect on the target variable.

In addition to the ANOVA test, other statistical tests such as the Chi-Square test and methods like mutual information can also be used for selecting K-best features. These methods are particularly effective in classification tasks and allow for the elimination of unnecessary or less impactful features in the dataset, thereby improving the overall performance of the model [16].

### 2.2. Conversion from Categorical to Numerical Data

The conversion of categorical data into numerical data is a critical step in data analysis and machine learning processes. This transformation allows categorical variables to be effectively used in statistical models and machine learning algorithms. Various transformation options and metrics used in this process must be carefully selected to preserve the accuracy of the data and minimize information loss [17]. In this section, the median function can be used in the transformation process, as it is unaffected by outliers and accurately reflects the central tendency of the data [18]. The median represents the middle value in a sorted dataset and is particularly useful in converting categorical data to numerical data, especially in ordered categories. It provides a reliable transformation for the numerical representation of ordered categories. Mathematically, the median is denoted as $\tilde{X}$ and is defined as shown in Equation (2).

(2) $$M=\arg\max_{x}f(x)$$

Here, *n*, represents the number of observations, and *x* represents the ordered dataset. In this study, the median is used because it accurately provides the central points of ordered values such as low, medium, and high for each feature in the dataset.

#### Fleiss’ Kappa

Fleiss’ Kappa is a statistical method used to measure the consistency between multiple raters or metrics. It is particularly used to assess the agreement between multiple evaluations made on the same dataset. This method accounts for the possibility of random agreement and evaluates the true agreement between metrics, providing a reliable comparison [19].

The Fleiss’ Kappa statistic is calculated by comparing the observed agreement $\left(\bar{P}\right)$ with the expected agreement $\left(\bar{P_{e}}\right)$. These ratios are calculated using the formula provided in Equation (3).

(3) $$\kappa =\frac{\bar{P}-\bar{P_{e}}}{1-\bar{P_{e}}}$$

Here, $\bar{P}$ represents the observed agreement rate and measures the true agreement between metrics. $\bar{P_{e}}$ represents the expected agreement rate and indicates the probability of random agreement between metrics.

The observed agreement rate $\left(\bar{P}\right)$ is calculated as shown in Equation (4).

(4) $$\bar{P}=\frac{1}{n}\sum_{i=1}^{n}P_{i}$$

Here, $P_{i}$ is the agreement rate between metrics for the *i*-th observation. *n* represents the total number of observations.

The expected agreement rate $\left(\bar{P_{e}}\right)$ represents the probability that the metrics will randomly reach the same result and is calculated using the formula provided in Equation (5).

(5) $$\bar{P_{e}}=\sum_{k=1}^{m}p_{k}^{2}$$

Here, *m* represents the number of metrics or categories used. $p_{k}$ represents the general proportion for each category.

The Fleiss’ Kappa value ranges from −1 to 1. A positive kappa value indicates that the agreement between metrics is higher than random agreement, while a negative kappa value indicates that the agreement is lower than random. In this context, the *k* value can be interpreted as follows: a value between 0.00 and 0.20 indicates slight agreement, between 0.21 and 0.40 indicates moderate agreement, between 0.41 and 0.60 indicates good agreement, between 0.61 and 0.80 indicates very good agreement, and between 0.81 and 1.00 indicates excellent agreement.

### 2.3. Ensemble Learning

**Figure 1:**
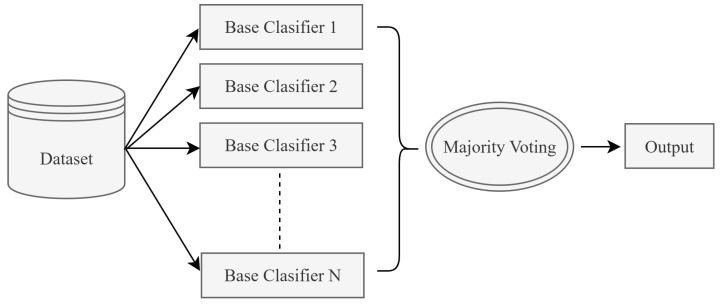
The post-model ensemble method (majority voting).

Ensemble learning is an effective and widely used approach that aims to increase the overall performance of a model by combining multiple machine learning algorithms. In this paper, the majority voting post-model ensemble method is applied. Majority voting is a method that enhances classification performance by making decisions based on the votes of multiple models [20]. Within the majority voting ensemble method, three fundamental classification algorithms are used together to generate final predictions through the majority voting approach in the ensemble learning structure. For each test sample, the classification results of these three algorithms are evaluated separately. The class label that appears most frequently (majority) is assigned as the final prediction. Thus, the goal is to obtain a more stable and generalizable model by combining the strengths of individual algorithms. The general structure of the majority voting ensemble is shown in Figure 1.

#### 2.3.1. Decision Tree

Decision tree (DT) is a non-parametric supervised learning method used for classification and regression problems [21]. This method creates a model in the form of a tree by splitting the dataset based on the most significant feature values. The decision-making process typically relies on selecting the feature with the highest Information Gain or the lowest Gini Impurity. For classification problems, Information Gain is calculated as follows:

(6) $$IG\left(T,A\right)=E\left(T\right)-\sum_{v\in Values(A)}\frac{|T_{v}|}{\left|T\right|}\times E\left(T_{v}\right)$$

Here, *T* represents the entire dataset, *A* represents the feature being examined, and $T_{v}$ represents the subset of the data where the value of feature *A* is *v*. E(T) represents entropy and is defined as follows:

(7) $$E\left(T\right)=-\sum_{i=1}^{n}p_{i}\log_{2}\left(p_{i}\right)$$

In this equation, $p_{i}$ represents the proportion of examples belonging to class *i*. The decision tree continues to split the dataset until a certain stopping criterion is met (such as maximum depth or minimum number of examples in a leaf).

#### 2.3.2. Random Forest

Random Forest (RF) is a non-parametric algorithm developed by Breiman that is widely used for classification and regression [22]. It makes predictions by combining multiple decision trees without requiring assumptions, and reduces overfitting.

Individual decision trees are often prone to overfitting. To address this issue, multiple trees are created using different subsets of data obtained through the bootstrap method. Ho’s random subspace method was adapted by Breiman into the RF algorithm [23].

The RF algorithm works as follows:

- Bootstrap samples are taken from the training data. For each sample, the best split is made from a randomly selected subset of features at each node. This continues until the minimum node size is reached. Once all the trees are constructed, their results are combined to make a prediction.
- As the number of trees increases, the error rate generally decreases. The size of the feature subset is a critical parameter for model performance.

#### 2.3.3. K-Nearest Neighbors

In K-Nearest Neighbors (KNN) classification, the distance to all examples in the dataset is calculated. However, during classification, only the *k* nearest neighbors to the target example are considered. These neighbors are the ones closest to the target point compared to all other examples. The *k* value is predetermined; a very small *k* can lead to the separation of similar examples into different classes, while a very large *k* can cause examples from different classes to be grouped together.

The KNN algorithm typically calculates the distance between the test example and all training data using a distance metric, such as Euclidean distance. Then, the *k* nearest neighbors are identified, and the most frequent class label among these neighbors is assigned to the test example. In the KNN algorithm, the training data (*X*), class labels (*Y*), and the *k* value are defined. For each example in the test data, distances to all examples in the training set are calculated. The *k* nearest neighbors are selected based on distance. The most frequent class label among these *k* neighbors is assigned to the test example. This process is repeated for all test examples to make predictions.

The performance of the KNN algorithm largely depends on the selected *k* value. Typically, the optimal *k* is determined experimentally [24].

### 2.4. Fuzzy Logic System

**Figure 2:**
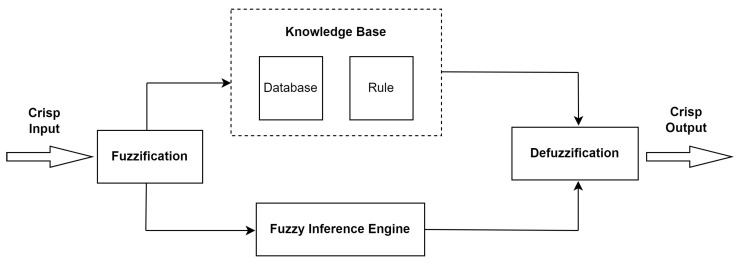
Fuzzy inference system.

Fuzzy logic emerged as an alternative approach to classical logic, enabling more precise modeling of complex systems based on human knowledge and expertise [25]. This approach allows working with uncertain data by considering ambiguities and uncertainties. Fuzzy inference systems (FISs), which are used for fuzzy reasoning, involve steps such as defining input and output variables, determining linguistic sets, defining membership functions, the fuzzification of precise input–output variables, creating a rule set, combining rule-set results, and the defuzzification of the final output [26]. The steps of FIS are shown in Figure 2.

In classical (non-fuzzy) set theory, an element either fully belongs to a set or does not [27]. For an element *a* in a conventional set *X*, the membership function $\mu_{X}\left(a\right)$ is defined as shown in Equation (8).

(8) $$\mu_{X}\left(a\right)=\left\{\begin{array}{cc} 1, & \;\mathrm{if}\;a\in X\\ 0, & \;\mathrm{if}\;a\notin X \end{array}\right.$$

This equation expresses that an element is either fully a member of the set ($\mu_{X}\left(a\right)=1$) or not a member at all ($\mu_{X}\left(a\right)=0$). However, in the fuzzy logic model, a system with multiple inputs and a single output is modeled using rules that contain multiple premises and a single result variable. Such a rule can be expressed as shown in Equation (9).

(9) $$\;\mathrm{Rule}\;R_{i}:\left\{\begin{array}{c} \;\mathrm{if}\;a_{1}\;\mathrm{is}\;X_{i1}\;\mathrm{and}\;\mathrm{if}\;a_{2}\;\mathrm{is}\;X_{i2}\;\mathrm{and}\;\ldots \;\mathrm{and}\;\mathrm{if}\;a_{n}\;\mathrm{is}\;X_{\mathrm{in}\;}\\ \;\mathrm{THEN}\;b\;\mathrm{is}\;Y_{i};i=1;2;\ldots ;m \end{array}\right.$$

Here, $X_{ij}\left(i=1,\ldots ,m,j=1,\ldots ,n\right)$ and $Y_{i}\left(i=1,\ldots ,m\right)$ are fuzzy sets. $a_{1},a_{2},\ldots ,a_{n}$ are the inputs, and *b* represents the output.

The rules and membership functions of fuzzy sets can be defined based on expert opinions or through accurate interpretation and clustering processes [28]. The two most commonly used models in fuzzy logic systems are the Mamdani and Sugeno models [29]. In the Mamdani model, both input and output variables are fuzzified, while in the Sugeno model, only the input variables are fuzzified. In the Mamdani model, the results of the rules are obtained as fuzzy sets, whereas in the Sugeno model, the results are crisp single values. In the Mamdani model, the centroid method is used to defuzzify the predicted fuzzy output, while in the Sugeno model, the final output is calculated using the weighted average method [30].

## 3. Proposed Method

**Figure 3:**
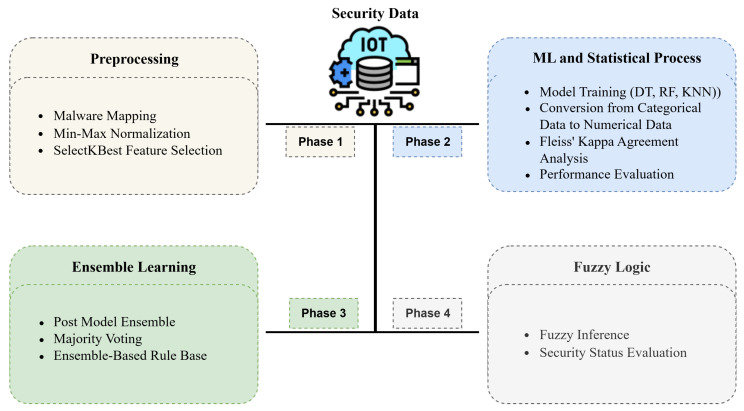
Overview of the proposed model.

This section presents the details of the proposed ensemble learning-based rule automation and fuzzy inference system for determining the IoT security level. The stages of the proposed model are illustrated in Figure 3.

### 3.1. Dataset Description

For the proposed ensemble learning-based rule extraction and fuzzy logic model, the ACI-IoT-2023 dataset, which contains IoT network traffic, is used. This dataset, developed by the Army Cyber Institute (ACI) for training and evaluating machine learning models for IoT network security, is a unique, realistic, and open access dataset [31]. Data collection was carried out in a simulated home environment within the IoT Research Laboratory at ACI. In this laboratory, both wired and wireless IoT devices, along with various automation systems, were managed using home assistant software. Network listeners strategically placed at key points recorded network traffic, capturing both normal and malicious activities. The dataset was divided into different types of data, such as labeled network flows, payload data, and raw packet captures, allowing for versatile analyses to be conducted using it [31].

The dataset contains a total of 85 columns, 84 features and 1 label, and over 1.2 million rows. The label includes benign and malware states. The malware types present in the dataset include Port Scan, ICMP Flood, Ping Sweep, DNS Flood, Vulnerability Scan, OS Scan, Slowloris, SYN Flood, Dictionary Attack, UDP Flood, and ARP Spoofing.

### 3.2. Preprocessing

A series of data-preprocessing steps are applied to analyze IoT-based network traffic data. These steps include removing missing and abnormal values from the dataset, organizing the labels, normalizing the dataset, and selecting specific features.

**Figure 4:**
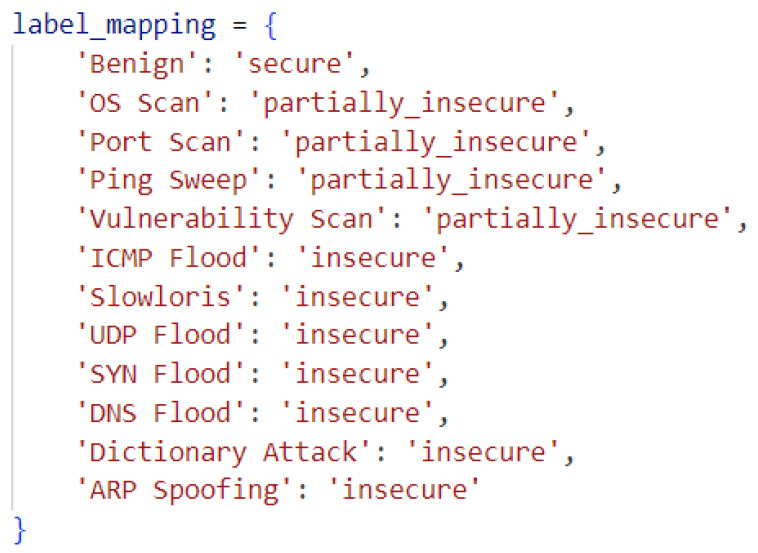
Label malware mapping.

The class labels in the dataset consist of attack types and benign traffic. These labels are reclassified as follows: benign traffic is labeled as “secure”, passive attacks (including scanning and eavesdropping) are labeled as “partially_insecure”, and active attacks (involving disruption, alteration, or interference with operations or data flow) are labeled as “insecure”. This malware-mapping process in the dataset is illustrated in Figure 4. Here, “Benign” represents normal traffic. “OS Scan”, “Port Scan”, “Ping Sweep”, and “Vulnerability Scan” correspond to the reconnaissance phase, which does not directly cause harm i.e., a potential threat. “ICMP Flood”, “Slowloris”, “UDP Flood”, “SYN Flood”, “DNS Flood”, “Dictionary Attack”, and “ARP Spoofing” represent explicit violation behaviors such as DoS, identity theft, spoofing, brute force attacks, or dictionary attack.

IoT-based network traffic data may sometimes contain missing or infinite values. In the dataset, infinite values are assigned NaN (Not a Number), and all NaN values are then replaced with the mean values of their respective columns. This process helps reduce potential model bias caused by missing data and ensures the completeness of the dataset. To control the influence of atypical records without employing an explicit anomaly detector, a conservative, label-agnostic preprocessing strategy is adopted. Following imputation, volatile fields that exhibit sparse or bursty behavior are discarded. The remaining numeric features are then coarsened into three bounded linguistic levels (low/medium/high) for use by the fuzzy layer, which caps the effect of extreme observations at bin boundaries and prevents single outliers from disproportionately affecting downstream steps. In addition, the subsequent ANOVA-based feature selection further de-emphasizes variables whose apparent separation is driven by idiosyncratic tails, yielding a more stable rule base.

Since there are dimensional differences among the features in the dataset, all features are normalized to a range between 0 and 1 using MinMaxScaler to facilitate the learning process of the model and to balance weight distribution during training. Subsequently, feature selection is applied to optimize classification performance. The ANOVA–based SelectKBest method evaluates the impact of each feature on classification. The four features that contribute most significantly to classification performance (“Idle Max”, “Fwd Seg Size Min”, “Flow IAT Min”, and “Flow IAT Mean”) are selected for use in the training process. “Idle Max” measures the duration IoT devices remain idle on the network, which may indicate the possibility of attackers staying passive for long periods before intrusion or to conceal an ongoing intrusion. “Fwd Seg Size Min” represents the minimum segment size of data packets and unusually small segment sizes can indicate potential attacks. “Flow IAT Min” and “Flow IAT Mean” measure the inter-arrival times between data packets. The minimum value reflects high data traffic intensity, while the mean value indicates the overall traffic speed of the network, providing insights into abnormal timings and possible attacks. These features are critical for detecting security threats in IoT networks at early stages. This is why the four selected features are not only statistically significant but also reflect the fundamental attack behaviors of IoT traffic.

In fuzzy rule-based classification systems, limiting the number of features improves interpretability and reduces rule explosion. Four features enable manageable rule generation while maintaining accuracy. In addition, this process reduces the size of the dataset, increases the inference speed of the model, and minimizes the impact of irrelevant features on classification performance. When each feature is defined with three linguistic terms (low, medium, high), the number of rules increases exponentially as $3^{K}$. With four features, the system generates $3^{4}=81$ rules, while with five features this number rises to $3^{5}=243$, and with six features it becomes $3^{6}=729$, continuing to grow exponentially. Four features provide a suitable balance by adequately representing domain knowledge and keeping the rule set at a manageable level, thereby preventing rule explosion in practical applications.

The dataset, after going through preprocessing steps, is tested using the majority voting method from ensemble learning models. The ensemble model consists of machine learning models with the highest accuracy and F-score rates. At this stage, the entire dataset is used for training, while the dataset prepared for fuzzy logic is used as the test set. For fuzzy, when all combinations of the three possible values (“low,” “medium,” and “high”) for four features are taken, an 81-row unlabeled test dataset is formed. To label these data, a series of preprocessing methods are applied to the test data. These are preprocessing steps to convert categorical data into numerical data. For this, the “low,” “medium,” and “high” boundaries are determined for each feature in the existing dataset, and the median value of each group is used to represent that group according to these boundaries. The main reason for using the median value is its robustness against outliers, its insensitivity to extreme values, and its ability to accurately reflect the central point of the data [18]. Thus, categorical data is transformed into numerical data and labeled by machine learning models as test data. Subsequently, the compatibility is tested, and final labels are determined with the ensemble model, leading to the creation of the rule base.

### 3.3. Automated Rule Extraction and Agreement Analysis Using Ensemble Learning

**Figure 5:**
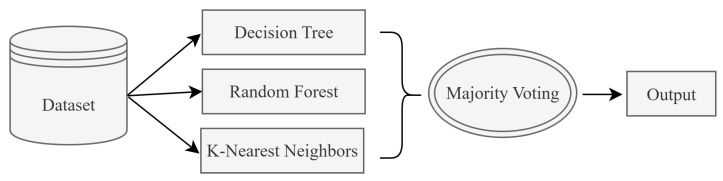
Post-model ensemble method (majority voting).

**Figure 6:**
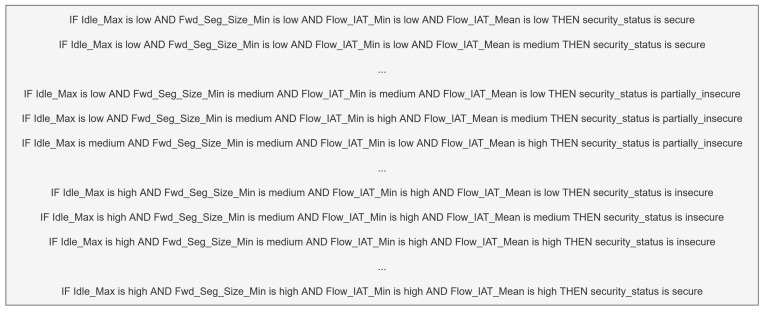
Fuzzy rule base obtained from ensemble learning.

Decision support for the fuzzy rule base is provided through an ensemble model that combines machine learning models. In this way, the fuzzy logic and ensemble model are used together to label the test data. The steps of the process are as follows:

- ML Accuracy Detection: After the dataset-preprocessing steps, the machine learning models are trained and their performance rates are compared. Among these methods are RF, DT, KNN, Gaussian Naive Bayes (NB), Logistic Regression (LR), and Support Vector Machine (SVM). The k-fold value is set to 10, and the performance rates of the classifiers selected for the ensemble model are evaluated. In k-fold cross-validation, each data point is used once as a test set and k − 1 times as part of the training set. Model performance is reported by averaging the results from k different runs. This ensures that the model is tested in a fairer, more reliable, and generalizable way. Some of the parameters used in this study are as follows: k-fold cross-validation with n_splits = 10, shuffle = True, and random_state = 42. KNN classifier with n_neighbors = 5 DT with criterion = ‘gini’ and random_state = 42. RF with n_estimators = 100, criterion = ‘gini’, and random_state = 42. LR with solver = ‘lbfgs’ and max_iter = 1000. For all models, the random_state value is fixed to ensure reproducibility of data splits, model initialization, and results. The performance rates of the models are shown in Table 2.
- As shown in Table 2, DT, RF, and KNN were chosen as the base classifiers. The reason is that, in our preliminary experiments, these three algorithms achieved higher performance compared to other candidates such as SVM, Naive Bayes, and Logistic Regression. Moreover, these algorithms provide a good balance between predictive accuracy and interpretability, which ensures compatibility with the fuzzy rule extraction step. DT produces transparent decision rules, RF reduces overfitting and improves stability through bagging, and KNN captures local neighborhood patterns that are particularly useful at class boundaries. The complementary nature of their error patterns made it appropriate to use these three methods together in the ensemble.
- Statistical Transformations: After determining the performance rates of the ML methods, the actual test data is generated. A categorical dataset consisting of 81 rows, created from combinations of low, medium, and high for four features, is constructed. The models are trained with numerical data, but the test data consists of categorical values. Therefore, the categorical data needs to be numerically transformed. For each feature, the boundaries corresponding to low, medium, and high states under a balanced distribution are determined as shown in Table 3. Based on these boundaries, the categorical values within the relevant range for each feature are represented numerically by the median value of that group. The median is robust against outliers and, especially in skewed distributions, best represents the class center [18]. The median values for each feature are shown in Table 4. In this way, a numerical dataset is created for the test data.
- Labeling Test Data with ML Models: The test data, converted from categorical to numerical values, is labeled separately with the highest-performing DT, RF, and KNN models. In this way, decision support is provided using machine learning methods to determine the label of the system for each feature’s low, medium, and high.
- Fleiss’ Kappa Compatibility Analysis: The Fleiss’ Kappa compatibility analysis method is used to test the agreement between the labels provided by the DT, RF, and KNN models for the test data. The agreement between the three classifiers for the same dataset is 0.8175949557123559. This indicates that the decisions made by the three classifiers, which have high accuracy rates, exhibit superior compatibility.
- Application of Majority Voting from Post-Model Ensemble Learning Methods: To improve the labeling decisions of the DT, RF, and KNN classifiers with the highest accuracy values, the majority voting method is applied as a post-model ensemble between these three classifiers. This method is shown in Figure 5.
- Obtaining the Fuzzy Rule Base Based on Ensemble Learning: Using the post-model ensemble method, majority voting is applied to the decisions made by the DT, RF, and KNN base classifiers, and the final decision is determined based on the majority vote. In this way, 81 data points consisting of combinations of low, medium, and high for the features are labeled. Each row in this dataset is defined as a rule base. An example of the obtained rule is shown in Figure 6.

### 3.4. Fuzzy Logic

In this paper, a fuzzy logic system is designed and implemented to evaluate the security status based on specific network traffic features. The key components of the fuzzy system include triangular membership functions, heuristic rule sets, and the Mamdani inference method.

**Figure 7:**
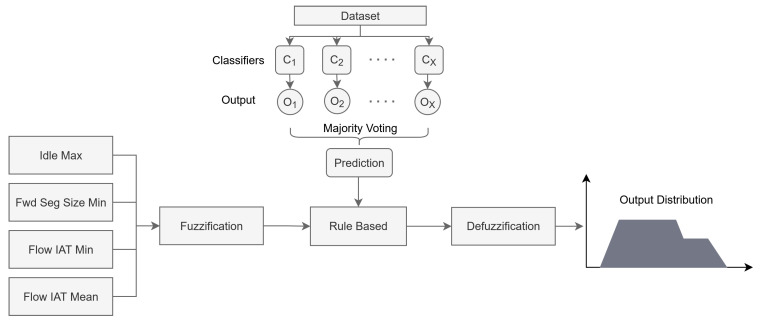
Proposed fuzzy decision support system with ensemble-based rules.

For the proposed fuzzy model, the rule base is automatically obtained by applying the majority voting method from ensemble learning, using multiple classifiers. The fuzzy inference system operates according to these rules. The obtained rule base is shown in Figure 6. The proposed fuzzy system is presented in Figure 7.

Following the dataset-preprocessing steps, fuzzy sets (low, medium, high) are defined for four numerical features (Idle Max, Fwd Seg Size Min, Flow IAT Min, Flow IAT Mean). For each feature and security status, the universe range is determined using the min–max values, and triangular membership functions (trimf) are assigned. Three membership functions are defined for each input variable and security status. The input variables are categorized as low, medium, and high, while the labels for the security status output are secure, partially insecure, and insecure. The universe range is statistically determined using the percentiles in Equation (10).

(10) $$q_{1}=P_{17},\;q_{2}=P_{33},\;q_{3}=P_{50},\;q_{4}=P_{67},\;q_{5}=P_{83}$$

The triangular output functions are as shown in Equation (11).

(11) $$\begin{array}{cc} \mu_{\mathrm{secure}}\left(x\right) & =\mathrm{trimf}(x;\min,\min,q_{2})\\ \mu_{\mathrm{partially}\_\mathrm{insecure}}\left(x\right) & =\mathrm{trimf}(x;q_{1},q_{3},q_{5})\\ \mu_{\mathrm{insecure}}\left(x\right) & =\mathrm{trimf}(x;q_{4},\max,\max) \end{array}$$

**Figure 8:**
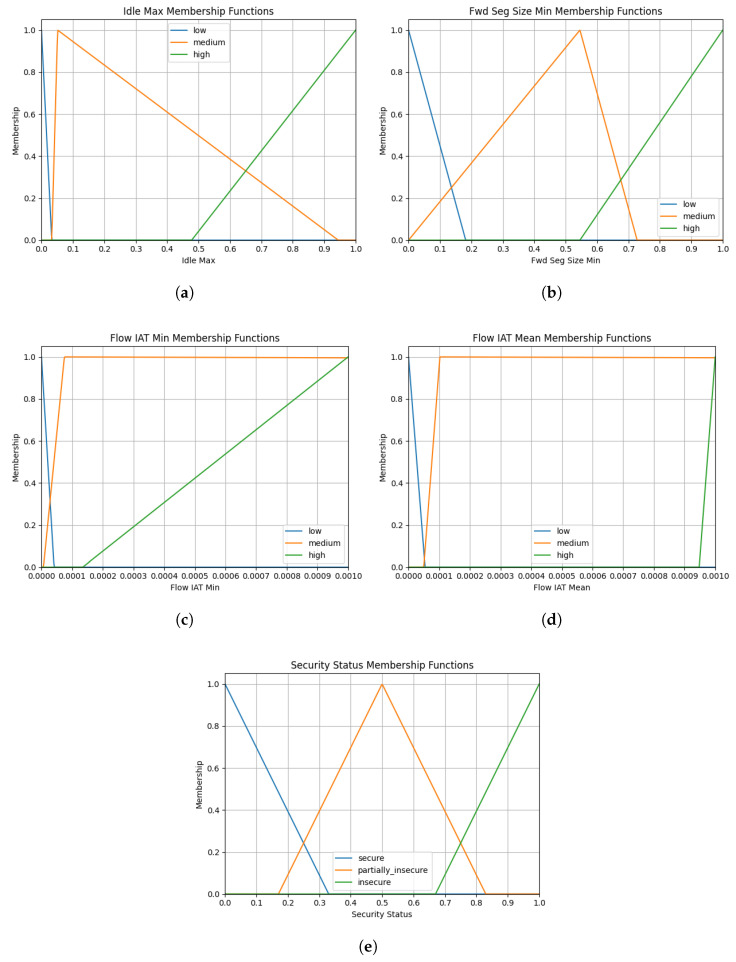
The plots of membership functions: (**a**) Idle Max. (**b**) Fwd Seg Size Min. (**c**) Flow IAT Min. (**d**) Flow IAT Mean. (**e**) Security Status.

When the membership function values for each input and output in the fuzzy system are determined according to Equation (10), the values shown in Table 5 are obtained. These values are applied as shown in Equation (11) to obtain the triangular membership functions. The graphs of these membership functions are shown in Figure 8.

In the fuzzy simulation system, the predicted security status *C* of the system for the security status score s∈[0,1] is expressed as follows:

$$C\left(s\right)=\left\{\begin{array}{cc} \mathrm{secure}, & s<0.33\\ \mathrm{partially}\_\mathrm{insecure}, & 0.33\leq s<0.67\\ \mathrm{insecure}, & s\geq 0.67 \end{array}\right.$$

One of the main advantages of the fuzzy logic system is its ability to present outputs in a graded manner rather than in sharp classes. For example, instead of simply labeling a packet as “secure” or “insecure,” the system can express the membership degrees of the relevant example to both the “secure” and “partially_insecure” sets. This approach, unlike traditional machine learning or threshold-based methods, offers decision-makers a more flexible and human-intuitive evaluation. Especially in areas like cybersecurity, where gray areas are often encountered, being able to indicate that a situation is not entirely secure but carries some degree of risk allows the system to be interpreted more meaningfully by security experts. Thus, through fuzzy inference, a warning such as “it might be partially insecure” can be issued, enabling more effective planning of proactive security measures.

## 4. Results and Discussion

This section presents the test environment, results, and evaluation of the proposed fuzzy security model based on ensemble learning–derived rules.

### 4.1. Experimental Setup

The implementation of the proposed model was performed on a computer equipped with 16 GB RAM, 12th Gen Intel(R) Core(TM) i7-12700H (20 CPUs) ∼2.3 GHz processor, and the Windows 11 operating system. The model is implemented in Python 3.11 using the sklearn, skfuzzy, numpy, statsmodels, and pandas libraries.

### 4.2. Preprocessing Results for Proposed Ensemble Model

Preprocessing steps such as feature selection, data normalization, and handling of missing or infinite values play a critical role in making the dataset suitable for ensemble learning and fuzzy inference. These preprocessing steps are particularly important when working with complex IoT data. Anomalies, missing values, and scale differences among features can significantly impact the performance of machine learning models. Using the SelectKBest method for feature selection ensures that only the most important features are included in the training process, thereby optimizing both computational efficiency and model performance.

The proposed approach, incorporating models such as DT, RF, and KNN, demonstrates strong performance with accuracy rates 99%. These models provide valuable insights for classifying the security status of IoT systems as “secure,” “partially_insecure,” and “insecure.” The ensemble method combines the strengths of each classifier while compensating for their weaknesses, thereby enhancing the overall reliability of the model. In particular, RF delivers balanced performance by effectively distinguishing security states, whereas the KNN model tended to misclassify at class boundaries due to its sensitivity to certain instance densities. However, the overall accuracy of the ensemble model, implemented using the majority voting method, remains high, making it a promising solution for IoT security monitoring. Fleiss’ Kappa analysis confirms excellent agreement among the three classifiers used in the ensemble model, with a value of 0.82. Furthermore, the proposed model provides both accuracy and flexibility in classifying IoT network traffic, thereby supporting comprehensive security assessments.

Unlike traditional binary classification models, fuzzy logic can assess security status in a more flexible manner. For instance, indicating that a device is not entirely secure but also not completely compromised. This is particularly valuable in IoT environments, where security threats often create ambiguous situations. Users gain not only a label but also an intuitive understanding of the confidence level associated with that label. This capability enables more meaningful and proactive security measures compared to traditional machine learning or fixed threshold-based methods.

### 4.3. Automated Rule Base

**Figure 9:**
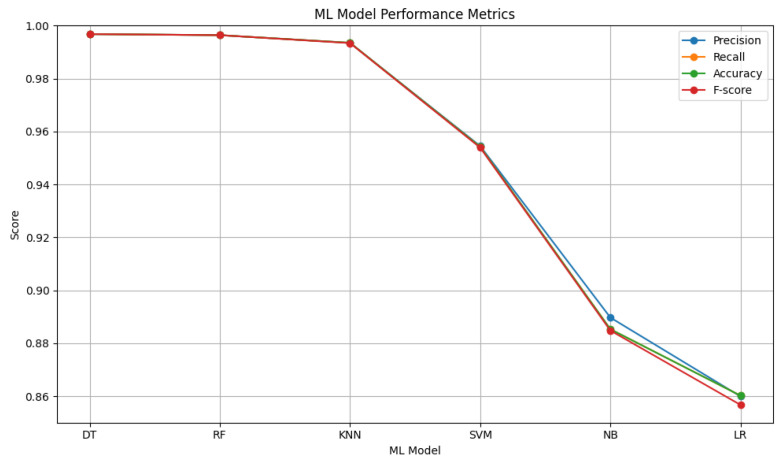
ML comparison for the proposed ensemble model.

For the proposed fuzzy security model based on ensemble-derived rules, machine learning models are tested. The comparative performance plot presented in Figure 9 illustrates the performance of six different machine learning algorithms evaluated in terms of four key metrics (accuracy, precision, recall, and F1-score). The DT, RF, and KNN models stand out clearly from the others by achieving high scores across all metrics. These three methods are particularly notable for their accuracy and F1-scores, approaching 0.99. In this context, the final classification is carried out using the majority voting method as a post-model ensemble approach applied to these selected models. This ensures that the strengths of each model are preserved while their weaknesses are balanced. SVM, on the other hand, shows moderate performance, being less effective than DT and RF in separating nonlinear patterns. Meanwhile, models such as NB and LR perform less successfully, particularly in this problem, which requires complex multi-class discrimination. These results indicate that selecting a model suited to the problem plays a decisive role in performance, and that decision tree-based methods are more favorable for security classification.

**Figure 10:**
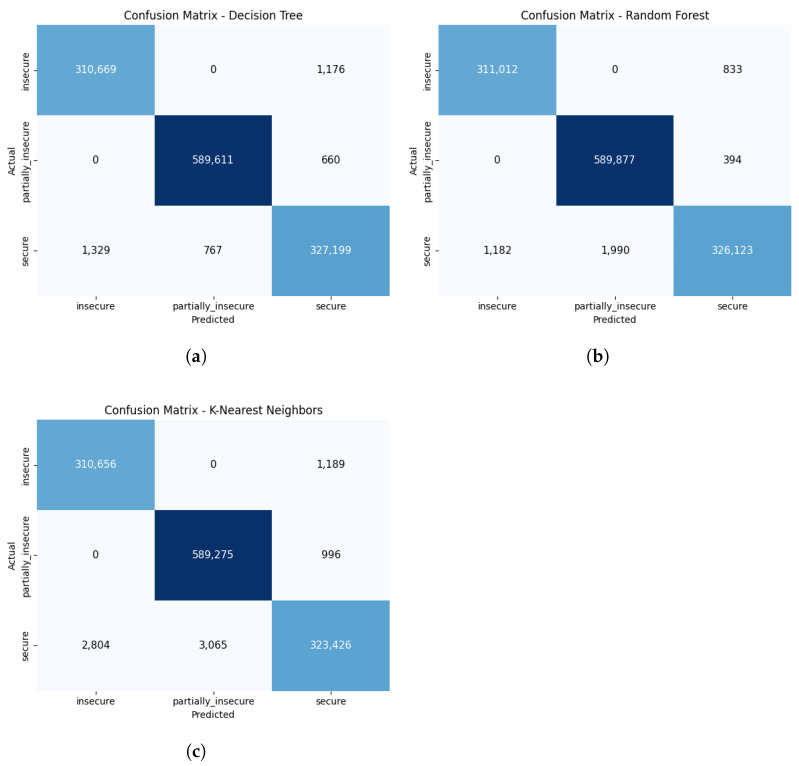
Confusion matrices of the ML models selected for the ensemble: (**a**) DT. (**b**) RF. (**c**) KNN.

The confusion matrices of the three ML methods with the highest accuracy rates selected for the ensemble are presented in Figure 10. The DT model generally demonstrates high accuracy in the “insecure” and “partially_insecure” classes, thanks to its distinct rule-based separation structure. However, a limited number of deviations occur in the “secure” class. The RF model, leveraging the collective output of numerous decision trees, effectively distinguishes between the “insecure” and “secure” classes, achieving a balanced distribution. The KNN model, on the other hand, tends to label instances in the “secure” class as “insecure” or “partially_insecure” when they are near class boundaries. This can be explained by the algorithm’s sensitivity to sample densities. These evaluations reflect the complementary strengths and weaknesses of the models, forming the main rationale for their combined use in the ensemble approach. The diversity in decision-making structures from each model merges within the majority voting method, thereby increasing overall accuracy and delivering a more robust classification performance.

The test dataset, prepared based on combinations of inputs, is labeled according to this ensemble learning model, and the rule base is generated. In other words, the decision-makers for the rules are multiple machine learning models, and the final decision is obtained by ensembling these models through majority voting.

### 4.4. Fuzzy Logic Results

Using the proposed ensemble model–based decision support method, the fuzzy rule base is generated, and fuzzy logic is applied. The inputs provided to the fuzzy system and the prediction score values obtained from the fuzzy system are presented in Table 6.

**Figure 11:**
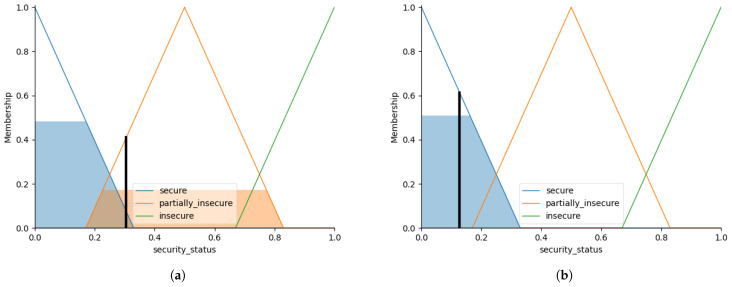

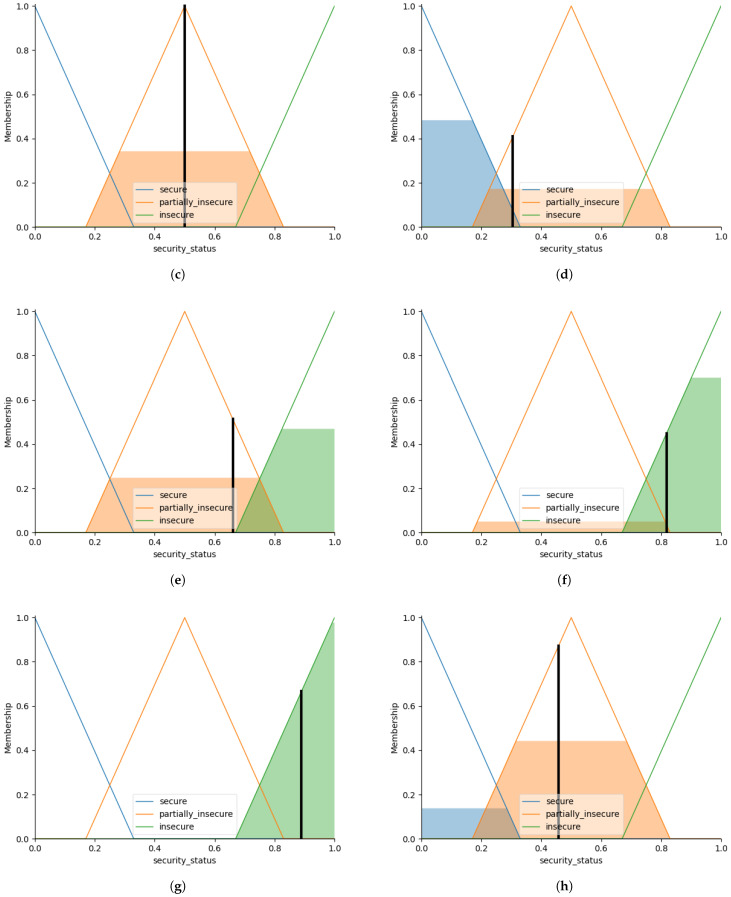
Fuzzy security status: (**a**) secure (0.305145532867). (**b**) secure (0.127784707465). (**c**) partially_insecure (0.500000000000). (**d**) secure (0.305009254350). (**e**) partially_insecure (0.660724529715). (**f**) insecure (0.817686940117). (**g**) insecure (0.889943930530). (**h**) partially_insecure (0.457817362860).

The fuzzy result graphs for the security status based on the values in Table 6 are provided sequentially in Figure 11.

Each graph in Figure 11 illustrates how the security score corresponding to the inputs intersects with the three membership functions. For example, in (a), the “secure” membership function is noticeably high, while the membership degree of the “partially_insecure” class remains lower. Similarly, in (e), a transition is observed between “partially_insecure” and “insecure,” indicating that the system generates multi-class membership contributions at boundary values. In this way, the user gains not only a label but also an intuitive sense of the confidence level associated with that label. The visualization demonstrates that fuzzy logic not only makes decisions but also transparently presents the reasoning behind those decisions.

One of the main reasons why fuzzy logic systems stand out in such security assessment applications is their ability to account for gray areas, unlike traditional classifiers. The system smoothly handles uncertainties, outliers, or gradual transitions in the data, preventing misclassifications that may arise from rigid thresholds. This approach offers significant advantages in terms of interpretability.

Table 7 demonstrates how the proposed fuzzy system enhances interpretability for end users by providing not only crisp security labels but also meaningful membership-based insights. Each example in Figure 11 is associated with specific input values, a fuzzy security score, and the final decision class, which together enable analysts to understand the degree of risk rather than relying on binary outcomes. For instance, a device categorized as “partially insecure” at the boundary between secure and insecure states signals a gray area that requires closer monitoring, while higher insecurity scores clearly suggest immediate isolation actions. In this way, the fuzzy membership outputs serve as an intuitive decision support tool, allowing security experts to prioritize responses and manage IoT threats more effectively.

### 4.5. Ablation Study and Computational Cost

The ablation study provides an opportunity to separately evaluate the contributions of the two core components of the proposed framework ensemble learning and fuzzy logic. Ensemble-based machine learning models deliver high accuracy and consistent outputs, enabling a firm labeling of the security state. However, such a rigid classification approach can offer limited information in borderline cases. In contrast, the fuzzy logic system, as illustrated in Figure 11, leverages the intersections of membership functions to produce a more flexible and graded evaluation. This feature not only yields a single class assignment but also presents more intuitive outputs that reflect different dimensions of the security status, thereby guiding potential actions as exemplified in Table 7. In this respect, the fuzzy logic approach provides more valuable support for security analysts in their decision-making processes.

The contribution of machine learning lies in transforming labeled IoT traffic from real-world environments into a rule base that can be utilized for decision support. This ensures that the fuzzy inference mechanism is built on a reliable and consistent foundation, resulting in a flexible structure applicable to different ecosystems. The ablation results presented in Table 8 show that the same eight test samples were labeled through ensemble voting as “secure, secure, partially_insecure, secure, insecure, insecure, insecure, insecure.” In contrast, the fuzzy system considered intermediate membership degrees for the same data, thereby producing a more detailed risk profile. This demonstrates that while the ensemble model provides robust hard-label decisions, the flexibility offered by fuzzy logic adds a dimension that is more closely aligned with human intuition in decision-making processes. Thus, the joint use of both approaches reinforces the overall strength of the proposed framework by combining measurable accuracy with interpretability.

The results presented in Table 9 demonstrate the computational costs of the proposed four-stage approach. The first three stages (I: preprocessing, II: ensemble model training and testing, III: rule base extraction) incur relatively higher time and memory costs; however, these processes occur during system setup and model preparation, rather than during the operational phase of IoT devices. For example, for the largest dataset fraction (100%), preprocessing was completed in 19.75 s, ensemble model training and testing took 158.94 s, and rule base extraction required 171.08 s. In contrast, CPU usage in these three steps remained within the 3–11% range, and memory consumption stayed at levels manageable for modern computer systems. Therefore, these costs do not create a direct burden in IoT environments and can instead be considered as an upfront investment at the initialization stage.

The most critical component, Stage IV (fuzzy inference), directly demonstrates the suitability of the model for IoT systems. Fuzzy inference was executed ten times for each dataset fraction and averaged. As shown in Table 9, the execution times remained between 0.124–0.143 s, memory usage stayed below 1 MB, and CPU utilization ranged from 4–6%. These values indicate that real-time security decisions can be achieved even on resource-constrained IoT devices. Thus, fuzzy inference is proven to be highly cost-effective and sustainable, reinforcing the practical applicability of the proposed approach in IoT environments.

The results in Table 10 further validate the performance of the majority voting ensemble model across different dataset fractions. In all cases, accuracy, precision, recall, and F-score values exceeded 99.4%, reaching as high as 99.67% on the full dataset. These strong performance outcomes enhance the reliability of the rule base extraction process, while the associated costs do not propagate into the fuzzy inference stage, thereby increasing the system’s overall advantage. In other words, the combination of a highly accurate rule base with a lightweight fuzzy inference process results in a hybrid solution that provides both accuracy and interpretability. This outcome represents one of the most significant findings, supporting both the theoretical contribution and the real-world applicability of the proposed method for IoT security.

This paper focuses on malware detection and security assessment within IoT ecosystems. The current experimental setting does not involve indoor localization benchmarks or ISO/IEC 18305 metrics. Although the proposed framework demonstrates adaptability across heterogeneous IoT ecosystems in terms of computational cost, it does not currently incorporate these standards. The integration of such evaluation methodologies requires specialized infrastructures and multimodal data collection pipelines. However, recent studies have shown that advanced indoor localization techniques, such as deep learning-based multiview BLE positioning [32] and multimodal approaches for smart homes evaluated under ISO/IEC 18305 metrics [33], can significantly enhance accuracy and robustness in location-aware IoT applications. Therefore, the incorporation of ISO/IEC-18305 driven indoor localization remains a promising future research direction for extending our hybrid ensemble–fuzzy framework toward more context-aware and spatially integrated IoT security solutions.

## 5. Conclusions

The security of IoT systems presents significant challenges, particularly due to their heterogeneous and dynamic structures. IoT devices often have limited resources and are frequently lacking robust security measures, making them vulnerable to various cyber threats. In this context, security solutions developed should not only be capable of accurate detection but also offer a flexible security assessment. The integration of ensemble learning and fuzzy logic proposed in our study demonstrates a comprehensive approach to meet these requirements. Ensemble learning enhances model accuracy, while fuzzy logic automates the rule base of the fuzzy system. Fuzzy logic provides the ability to evaluate and interpret the security status of IoT networks in greater detail, enabling better decision-making in uncertain and complex threat environments.

In conclusion, the integration of ensemble learning and fuzzy logic systems represents a significant advancement in the field of IoT security. This approach provides a multi-layered and flexible solution for ensuring IoT security, making it highly promising for real-world applications. Particularly, it enables more effective decision-making in uncertain and gray areas while securing IoT systems. This offers an effective solution for security management in IoT systems. Although the experiments were conducted on a single dataset, an ablation study across different dataset fractions is additionally performed to examine the scalability of the proposed framework. The results confirmed that both performance and computational efficiency were preserved under varying data volumes.

Future studies can extend this evaluation to heterogeneous IoT environments with diverse devices and attack scenarios, with a particular focus on enabling the fuzzy system to handle more complex threats and increasing the adaptability of the framework to broader IoT settings. Beyond these directions, future work can also address real-time adaptability by allowing the rule base to evolve with dynamic threat landscapes. The lightweight structure of the fuzzy inference stage highlights the feasibility of deploying the framework on edge devices with limited computational resources. Moreover, incorporating privacy-preserving mechanisms would further enhance applicability in sensitive IoT domains. Potential use cases include intrusion detection in smart city infrastructures, anomaly monitoring in industrial IoT, and adaptive security management in smart homes and healthcare systems. In particular, the interpretability of fuzzy outputs can support security analysts in identifying potential anomalies at an early stage, enabling proactive monitoring and timely interventions.

## Figures and Tables

**Table 1:** Review of related works (ordered by year).

Ref.	Year	Description	Method	IoT Area	Results
[6]	2020	Routing protocol for IoT	Fuzzy Logic, Multi-Fuzzy Model, Trust Model	IoT Routing Security	Improved network performance and attack detection
[5]	2020	Routing optimization in IoT	Fuzzy Logic, Reinforcement Learning	IoT Routing Optimization	Improved network lifetime and energy efficiency
[7]	2021	Security assessment in fog-based IoT	IVIFS-AHP, MCDM	Fog-IoT Security	Prioritization of security factors
[10]	2022	Security mechanism for detecting malicious behavior	Fuzzy Logic, Fog Computing, Zero-Trust Policy	Fog-IoT Security	High accuracy, low resource overhead
[9]	2023	Attack detection in IoT	Fuzzy Neural Network, SDN	IoT Security	Enhanced attack detection accuracy
[11]	2023	Security risk assessment in industrial IoT	Fuzzy Inference System, Linguistic Variables	IIoT Security	Improved decision support
[8]	2023	Secure architecture for IoT	Fuzzy Logic, Fog Computing, DDoS Prevention	IoT Security	Enhanced real-time attack detection accuracy
[3]	2023	Cyber defense security for next-gen IoT	Fuzzy rule-based classifiers, Machine learning	CIoT	High web spam detection accuracy
[4]	2023	Threat detection in IoT Networks	Fuzzy DL, Optimized ANFIS, Fuzzy Matching	Blockchain-based IoT Security	Superior performance in accuracy and F1-score
[12]	2024	Hybrid attack detection system for IoT networks	Fuzzy Logic, Decision Tree, Clustering	IoT Security	High accuracy, performance, and detection success
[13]	2025	Secure relay selection algorithm	Fuzzy Logic, Tabu Algorithm	WSN Security	Energy efficiency, reduced retransmissions, isolation of malicious nodes
[14]	2025	Intrusion detection for IoT	Fuzzy Logic, CNN, Entropy, Feature Selection	IoT Security	Successful attack detection, reliable classification results

**Table 2:** Comparison of the performance of machine learning methods.

ML Model	Precision	Recall	Accuracy	F-Score
DT	0.9968	0.9968	0.9968	0.9968
RF	0.9964	0.9964	0.9964	0.9964
KNN	0.9935	0.9935	0.9935	0.9934
SVM	0.9545	0.9542	0.9542	0.9539
NB	0.8897	0.8853	0.8853	0.8848
LR	0.8599	0.8602	0.8602	0.8567

**Table 3:** Boundary (B) values for each feature.

Features	B (Low)	B (Low–Medium)	B (Medium–High)	B (High)
Idle Max	0.0	0.0327408205	0.4778552243	1.0
Fwd Seg Size Min	0.0	0.1818181818	0.5454545454	1.0
Flow IAT Min	0.0	0.0000532440	0.0001277126	1.0
Flow IAT Mean	0.0	0.0000634909	0.0007005925	1.0

**Table 4:** Median values corresponding to each value of each feature.

Features	Low	Medium	High
Idle Max	0.0326968103818217	0.0484954050170927	0.4888663728675055
Fwd Seg Size Min	0.0	0.5454545454545454	0.7272727272727273
Flow IAT Min	0.0000054639068678	0.0000737378312857	0.0306466882538042
Flow IAT Mean	0.0000491087370405	0.0001005757563467	0.0830450854138886

**Table 5:** The triangular membership function values for fuzzy (according to Equation (10)).

Values	Idle Max	Fwd Seg Size Min	Flow IAT Min	Flow IAT Mean
min	0.0	0.0	0.0	0.0
q1	0.032698084930	0.0	6.4935792867 × 10^−6^	4.9573750445 × 10^−5^
q2	0.032717498164	0.181818181818	4.1020820558 × 10^−5^	5.3825301572 × 10^−5^
q3	0.051290879003	0.545454545455	7.4734288465 × 10^−5^	0.000102170088
q4	0.478048911682	0.545454545455	0.000134654580	0.000947735184
q5	0.944537272628	0.727272727273	0.207061827262	0.214860366937
max	1.0	1.0	1.0	1.0

**Table 6:** Fuzzy security status score for sample inputs.

Idle Max	Fwd Seg Size Min	Flow IAT Min	Flow IAT Mean	Security Status Score
0.006221688797	0.094042473326	0.000000000000	0.000000000000	0.305145532867
0.264933761292	0.776827809269	0.008247201600	0.018999679827	0.127784707465
0.021500476863	0.402127314907	0.005735505107	0.011737656402	0.500000000000
0.264295998754	0.093972995210	0.008219071188	0.506646538419	0.305009254350
0.723005723412	0.357606249770	0.500116286997	0.000501555938	0.660724529715
0.900000000000	0.500000000000	0.700000000000	0,000080000000	0.817686940117
0.999900000000	0.533000000000	0.999000000000	0.990000000000	0.889943930530
0.550000000000	0.500000000000	0.000250000000	0.002000000000	0.457817362860

**Table 7:** User-facing interpretability of fuzzy outputs (corresponding to Figure 11).

Figure 11	Input Values (Idle Max, Fwd Seg Size Min, Flow IAT Min, Flow IAT Mean)	Fuzzy Score	Fuzzy Decision	Security Analyst Insight
(a)	0.0062,0.0940,0.0000,0.0000	0.3051	Secure	Low risk. Routine monitoring is sufficient.
(b)	0.2649,0.7768,0.0082,0.0190	0.1278	Secure	Normal network behavior. No intervention required.
(c)	0.0215,0.4021,0.0057,0.0117	0.5000	Partially insecure	Gray area. Potentially suspicious traffic. Analyst should conduct additional log analysis.
(d)	0.2643,0.0940,0.0082,0.5066	0.3050	Secure	Mostly safe, but high traffic load detected. Monitoring is recommended.
(e)	0.7230,0.3576,0.5001,0.0005	0.6607	Partially insecure	Potential transition toward attack. Early warning. Anomaly monitoring should be increased.
(f)	0.9000,0.5000,0.7000,0.00008	0.8177	Insecure	Serious risk. Device should be quarantined or disconnected from the network.
(g)	0.9999,0.5330,0.9990,0.9900	0.8899	Insecure	High risk of security breach. Immediate isolation and incident response required.
(h)	0.5500,0.5000,0.00025,0.0020	0.4578	Partially insecure	Uncertain case. Analyst should further investigate abnormal traffic patterns.

**Table 8:** Ablation results comparing ensemble and fuzzy for the test samples.

Results	(a)	(b)	(c)	(d)	(e)	(f)	(g)	(h)
Fuzzy	Secure	Secure	Partially Insecure	Secure	Partially Insecure	Insecure	Insecure	Partially Insecure
Ensemble	Secure	Secure	Partially Insecure	Secure	Insecure	Insecure	Insecure	Insecure

**Table 9:** Computational costs of the comprehensive model for dataset fractions.

**xDataset**	**25%**			**50%**		
**Stage**	**Time (s)**	**Memory (MB)**	**CPU (%)**	**Time (s)**	**Memory (MB)**	**CPU (%)**
I	12.23	948.84	12.9	14.93	1044.69	9.9
II	47.03	77.20	5.5	107.77	31.78	4.3
III	53.95	84.13	6.2	95.21	147.49	6.0
IIV	0.135	1.02	5.01	0.124	0.95	4.09
**xDataset**	**75%**			**100%**		
**Stage**	**Time (s)**	**Memory (MB)**	**CPU (%)**	**Time (s)**	**Memory (MB)**	**CPU (%)**
I	17.85	1102.26	14.3	19.75	1056.17	8.4
II	130.57	170.66	3.3	158.94	67.64	7.2
III	145.95	191.7	8.8	171.08	240.02	11.4
IIV	0.143	0.85	5.33	0.128	0.86	5.88

**Table 10:** Performance metrics of the ensemble model for dataset fractions.

xDataset	Precision	Recall	Accuracy	F-Score
25%	0.9946	0.9946	0.9946	0.9946
50%	0.9955	0.9955	0.9955	0.9955
75%	0.9961	0.9961	0.9961	0.9960
100%	0.9967	0.9967	0.9967	0.9967

## Data Availability

The original contributions presented in this study are included in the article. Further inquiries can be directed to the corresponding author.

## Abbreviations

The following abbreviations are used in this manuscript:

ARP	Address Resolution Protocol
CPU	Central Processing Unit
CNN	Convolutional Neural Network
DDoS	Distributed Denial of Service
DNS	Domain Name System
IAT	Inter-Arrival Time
ICMP	Internet Control Message Protocol
MCDM	Multi-Criteria Decision Making
ML	Machine Learning
OS	Operating System
RAM	Random Access Memory
SYN	Synchronize (TCP flag)
UDP	User Datagram Protocol
WSN	Wireless Sensor Network
